# Spatial Analysis of Breast Cancer Mortality Rates in a Rural State

**DOI:** 10.5888/pcd19.220113

**Published:** 2022-10-20

**Authors:** Marisa Schulz, Emma Spors, Kari Bates, Semhar Michael

**Affiliations:** 1 Mathematics and Statistics Department, South Dakota State University, Brookings, South Dakota; 2 Sanford Research, Research Design and Biostatistics Core, Sioux Falls, South Dakota

## Abstract

**Introduction:**

Breast cancer affects 1 in 8 women in the US and is the most frequently diagnosed cancer in women. In South Dakota, 102 women die from breast cancer each year. We assessed which sociodemographic factors contributed to mortality rates in South Dakota and used spatial analysis to investigate how counties’ observed age-adjusted mortality rates compared with expected rates.

**Methods:**

We computed standardized incidence ratios (SIRs) of all counties in South Dakota by using the age-adjusted mortality rates, the 2000 US standard population, and the South Dakota estimated population. We used a linear regression model to identify sociodemographic factors associated with breast cancer mortality rates and to compute a new SIR value, after controlling for relevant factors.

**Results:**

Educational level and breast cancer incidence rates were significantly associated with breast cancer mortality rates at the county level. The SIR values based on age-adjusted counts showed which counties had more deaths due to breast cancer than what might be expected using South Dakota as the reference population. After controlling for sociodemographic factors, the range of SIR values decreased and had lower variability.

**Conclusion:**

The regression model helped identify factors associated with mortality and provided insights into which risk factors are at play in South Dakota. This information, in combination with the spatial distribution of mortality by county, can be used to help allocate resources to the counties in South Dakota that need them most.

**Summary**

**What is known on this topic?**

Breast cancer affects 1 in 8 women in the US and is the most frequently diagnosed cancer in women.

**What is added by this report?**

Because South Dakota is a rural state, sociodemographic factors affect the population differently than in the general US population. We assessed the spatial distribution of breast cancer mortality rates by county, and our findings add insight on educational attainment as a risk factor for breast cancer.

**What are the implications for public health practice?**

Our results can be used to help allocate resources to the South Dakota counties that need them most.

## Introduction

In South Dakota, breast cancer is the most commonly diagnosed cancer and the second leading cause of cancer death among women (1,2). In 2022, an estimated 750 new cases and 110 deaths attributed to female breast cancer will occur in South Dakota. In general, a woman in the US has a 1-in-8 lifetime risk of developing breast cancer (3,4). Since 1989, the US breast cancer mortality rate has decreased 40%, but from 2010 to 2019 the rate slowed to a low of decreasing by 1.3% per year (5).

Characteristics such as age and race and ethnicity affect a woman’s chances of being diagnosed with or dying of breast cancer, but new evidence has established that sociodemographic factors, including education level, also play a role (6). Albano et al noted a negative relationship between number of years of education and cancer mortality and found that the level of education and race vary considerably with mortality rates (7). Of the South Dakota population aged 25 years or older, 92.2% are high school graduates (higher than the national average) and 29.3% have a bachelor’s degree or higher (lower than national average) (8). Olson et al acknowledged that communities exist in which geographic disparities are more prominent because of rural isolation and small population size (9). Furthermore, 64 of 66 counties in South Dakota are categorized as rural or frontier, and South Dakota contains 9 American Indian reservations (10). Finally, 61.6% of women receiving breast services are White, and 16.7% are American Indian (11); most of the population in South Dakota is White, and the leading minority is 8.8% American Indian (8).

The study aimed to describe the spatial distribution of female breast cancer mortality at the county level in South Dakota and assess the association between mortality rates and risk factors reported in the literature.

## Methods

### Data source

The 66 counties of South Dakota have boundaries that are defined by the South Dakota Legislature and accepted by the US Census. The counties range in population from 183,439 in Minnehaha County to 917 in Jones County, and the median population per county is 5,413. Most residents of South Dakota were White; the median percentage of non-White residents by county was 6.6% and the maximum was 95.2%

### Cancer data

Breast cancer incidence and mortality rates from 2008 through 2017 were extracted from the South Dakota State Cancer Registry’s South Dakota Cancer County Assessment Tool (12). The tool allows access to public use cancer data. The 2008–2017 data were accessed in September 2020. Both rates were per 100,000 persons and age-adjusted to the 2000 US standard population and the South Dakota estimated population. The proportion of mammography screening rates in South Dakota was based on the numbers reported by Holzhauser et al for the All Women Count! mammography program (13). The average number of participants for 1997 through 2016 was reported for the program; then, the average number of participants was adjusted for the total number of women older than 40 years in the county to get an estimated screening rate for each county (13,14).

### Demographics

We used 2015 data from the US Census Bureau to obtain information on the 66 South Dakota counties, including the number of providers and the education level, poverty level, percentage of uninsured, median age, and race of residents (8).

Data on educational attainment were obtained from the US Census Bureau’s American Community Survey (ACS). These data were count estimates for the population of each county aged 25 years or older. Levels were categorized as less than 9th grade, 9th through 12th grade with no diploma, high school graduate or equivalent, some college but no degree, associate degree, bachelor’s degree, and graduate or professional degree. These values were modified into an educational attainment statistic of the percentage of the population with less than a bachelor’s degree of education. The statistic used in this study was the percentage of the population with less than a bachelor’s degree, by county.

We collected data on poverty estimates, by county, from the ACS; these data adhered to the standards specified by the Office of Management and Budget in Statistical Policy Directive 14 (8). Poverty was determined by a set of income thresholds that consider the living situation (alone or with nonrelatives), age, and number of people per household. For example, the poverty threshold for 2-person families varies by the age of the primary householder and differs from the poverty threshold for people living alone or with nonrelatives, which also varies by age. 

Insurance coverage percentages were collected from Small Area Health Insurance Estimates (SAHIE) (15). The uninsured percentage included residents who were not covered by insurance, which excluded those on government assistance such as Medicaid or Medicare. Finally, the data set summarizing racial distributions in a county included estimated population counts for American Indian and Alaska Native, Asian, Black or African American, Native Hawaiian or other Pacific Islander, White, other race, and 2 or more races. Because of South Dakota's predominantly White population, the data were configured into White and non-White, which determined the non-White percentage per county (8) (Table 1).

**Table 1:** Factors for Regression Analysis, Study on Breast Cancer Mortality in South Dakota, 2008–2017

Factor	Description
Breast cancer incidence rates	Age-adjusted breast cancer incidence rate per 100,000 persons
Breast cancer screening rates	Estimated mammography screening rates
Number of screening providers	The number of medical providers per county that provide breast cancer screening
Poverty level	The percentage of residents living in poverty per county
Insurance status	The percentage of residents without any insurance
Median age	Median age of the county’s residents
Race	The percentage of residents that are non-White
Educational attainment	The percentage of the population aged 25 years or older with less than a bachelor’s degree

### Statistical analysis

#### Data manipulation and missing value imputation

Mortality rates and the various independent variables were combined into 1 data set; 15 of the 66 counties were missing mortality rate data. Mortality rates are often suppressed from public availability when 3 or fewer deaths are reported in a county, to protect patient identity. To remedy the missing data, k-nearest-neighbor (KNN) imputation was used to estimate the missing mortality values. KNN imputation compares a data point *x_i_* with its *k* nearest neighbors and then approximates *x_i_* using the majority vote of these *k* neighbors in multidimensional space. For the data, *k* = 9 nearest neighbors were used, and a weighted mean of the *k* nearest values was placed for each missing *x_i _* (16,17). This was done with the function “knn()” from the R package VIM version 6.1.1 (R Foundation for Statistical Computing) (18).

#### Multiple linear regression

We used multiple linear regression to model the relationship between the factors in this study and breast cancer mortality rates (19). We considered several potential predictor variables with observed correlation, hence a stepwise variable selection technique was used, in both the forward and backward direction, to perform feature selection. As a result, a subset of the factors that were associated with the mortality rates was obtained based on Akaike information criterion (AIC) (19,20).

The resulting model is of the form $\hat{Y_{i\;}}=\;{\hat{\beta }}_{0}\;+\;{\hat{\beta }}_{1}X_{1i}+\ldots +{\hat{\beta }}_{p}X_{ji},$ for *i* = 1,2, … ,66 where $\hat{Y_{i\;}}$ represents the estimated mortality rate for the *i^th^* county and ${\hat{\beta }}_{0}$ is the intercept. The variables *X*_1_ *_i _* through *X_ji_* represent the values of the factors for the *i^th^* county and ${\hat{\beta }}_{1}$ through ${\hat{\beta }}_{p}$ are the coefficients that were estimated using the least squares regression method (19,21). To explore the data, 4 linear regression models were created, which differ by factors included in the model.

The first regression model included all 8 factors as prediction variables resulting in Model 1. The regression model was then fitted by stepwise variable selection in both the forward and backward direction by using AIC as a model selection criterion. AIC rewards goodness of fit and penalizes the model’s complexity (19). This was done by using the R package MASS version 7.3–54 (R Foundation for Statistical Computing) (22). The simplified model resulted in Model 2. At this point, a decision was made to remove the incidence rate from the data to better see how the other sociodemographic factors contributed to breast cancer outcomes, resulting in Model 3. Model 3 was then fitted with stepwise variable selection, yielding Model 4. To best compare the expected mortality to the observed mortality with all predictors available, Model 2 was chosen as the final model. Model 2 was then used to predict the expected mortality rates for the second standardized incidence ratio (SIR) that was computed (*SIR_LM_*).

### Standardized incidence ratio

A SIR was used to compare the spatial distribution of counties in terms of mortality rates due to breast cancer. In general, the SIR compares the expected value of deaths to the observed value of deaths in a county. This is calculated with $SIR_{j}=\frac{O_{j}}{E_{j}}$ where *O_j_* is the observed value for county *j*, and *E_j_* is the expected value for county *j*. A SIR of greater than 1 means that there were more deaths than expected, whereas a SIR of less than 1 means that there were fewer deaths than expected for that county.

Because age-specific mortality rates by county were not available, the observed age-adjusted counts per county were computed from the age-adjusted rates per county as follows: $O_{j}^{*}=\frac{O_{j}}{100,000}y_{j}$ and $E_{j}^{*}=\left(\frac{\sum_{j=1}^{66}O_{j}}{100,000}\cdot y_{j}\right)\cdot \frac{y_{j}}{\sum_{j=1}^{66}y_{j}}\;$, where *O_j_* is the *j^th^* county’s observed age-adjusted mortality rate and *y_j_* is the *j^th^* county’s population. The expected count was computed to be on the same scale as the observed count. These manipulations were used only to calculate an age-adjusted count SIR, referred to as *SIR_COUNT_*. All other analysis of the data was completed with the original variable (*O_j_*), as described in the data source.

A SIR was calculated on the mortality in each South Dakota county (N = 66). To account for the age adjustment of the data, 2 different SIRs were found: using the age-adjusted mortality count for South Dakota and using the expected rate obtained from the linear regression model. The first SIR accounted only for the age adjustment, and the second SIR accounted for more factors related to breast cancer. For example, the first SIR used the age-adjusted mortality count for a county for both observed and expected (*SIR_COUNT_*). The second SIR (*SIR_LM_*) used the mortality rates by county, which were per 100,000 persons and age-adjusted to the 2000 US standard population, and the South Dakota estimated population for observed and predicted mortality rate from the linear regression model for expected, which accounted for incidence rates and educational attainment. All statistical analyses used R version 4.1.2 and RStudio version 2021.09.2 build 382 (R Foundation for Statistical Computing) (23,24).

## Results

### Exploratory data analysis

**Figure 1:**
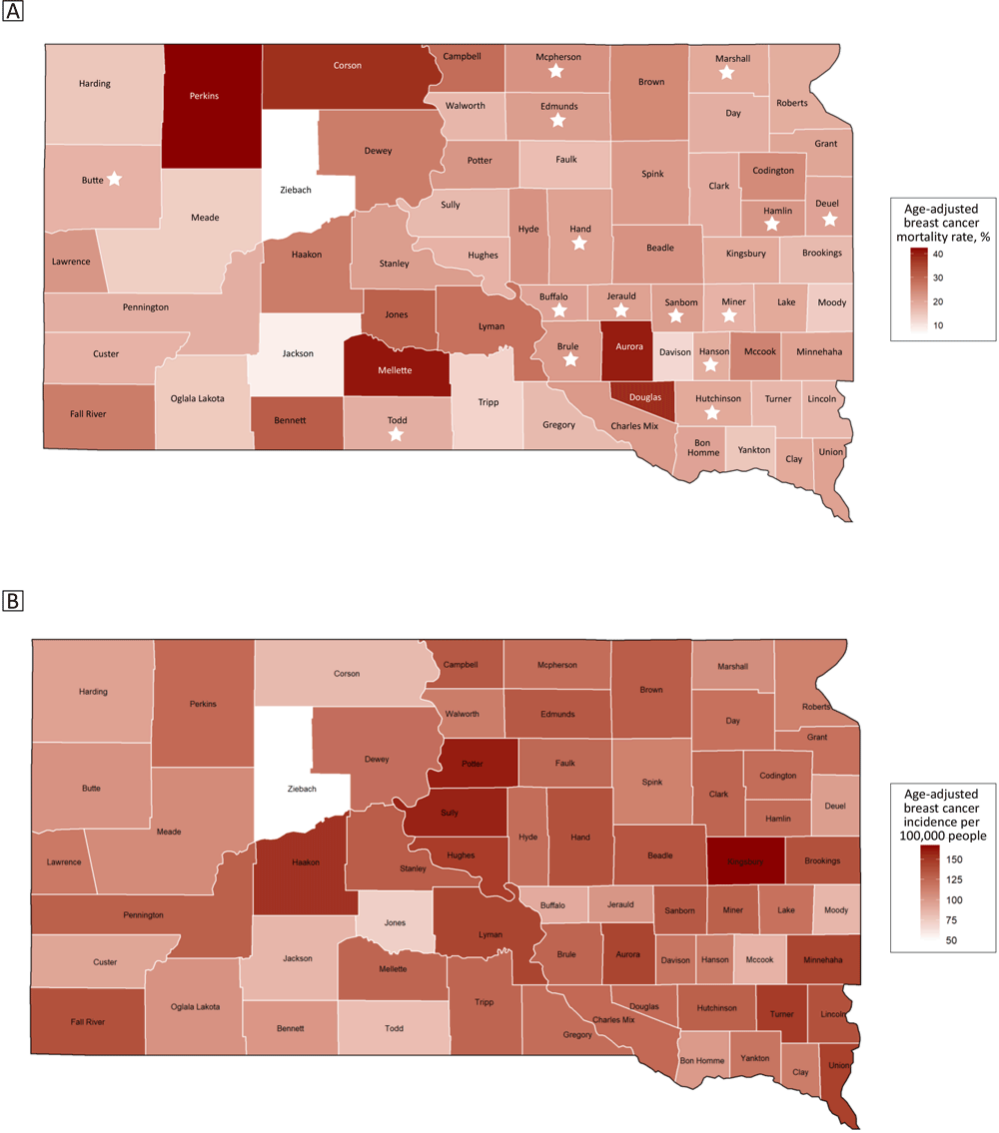
Map A shows the age-adjusted breast cancer mortality rates and Map B shows the age-adjusted breast cancer incidence rates, by county (N = 66), South Dakota, 2008–2017. Counties whose mortality rates have been imputed are marked with a star. Source: South Dakota State Cancer Registry, South Dakota Department of Health (12).

To learn more about the data, we performed an Exploratory Data Analysis (EDA) using several techniques. We explored the geographic distribution of the breast cancer mortality rates by using a choropleth map of South Dakota with age-adjusted mortality and incidence rates (Figure 1). The eastern side of the state had lower and more consistent mortality rates, followed by the far western part of the state. The central west part of the state exhibited higher mortality rates.

Five counties had higher mortality rates than the rest of the counties: Perkins, Mellette, Aurora, Douglas, and Corson. Two counties had lower mortality rates than the rest of the counties: Ziebach and Jackson. Eight counties (Bennet, Buffalo, Corson, Jackson, Mellette, Oglala, Todd, and Ziebach) had a poverty percentage greater than 30%, which was distinctly higher than the others; each county contains land on an American Indian Reservation. Minnehaha and Pennington counties had high screening rates; these counties are home to the first- and second-largest cities in South Dakota, respectively, so they also had the greatest number of screening providers.

All variables, besides the number of providers and screening rate, had a correlation greater than zero with mortality rate. Incidence, educational attainment, and uninsured percentage all had a low positive correlation with mortality rate. The highest correlation with mortality rate was education attainment, at a value of 0.26. The remaining variables had a negligible correlation with mortality rate.

### Regression analysis

Model 2 had the highest adjusted *R* ^2^ value (0.10) and the lowest AIC (441.79), with 2 significant factors associated with mortality rate; Model 3 had the lowest adjusted *R* ^2^ (.004) and the highest AIC (453.32), with no significant predictors of mortality. In Model 2, breast cancer incidence and educational attainment were predictors of breast cancer mortality, indicating that as more people are diagnosed with breast cancer and as the percentage of people with less than a bachelor’s degree increases, breast cancer mortality rate increases (Table 2). Educational attainment was a predictor of mortality in all models, and the educational attainment statistic had a *P* < .001. 

**Table 2:** Regression Models, Study on Breast Cancer Mortality in South Dakota, 2008–2017

Coefficient	Model 1	Model 2	Model 3	Model 4
	Estimate (95% CI)			
Intercept	−15.375 (−39.388 to 8.638)	−11.113 (−32.253 to 10.026)	−2.248 (−23.425 to 18.929)	4.114 (−12.158 to 20.386)
Median age	−0.059 (−0.481 to 0.363)	—	−0.043 (−0.478 to 0.391)	—
Non-white percentage	−15.119 (−37.725 to 7.486)	—	−11.266 (−34.233 to 11.700)	—
Poverty percentage	0.208 (−0.292 to 0.708)	—	0.030 (−0.455 to 0.515)	—
Uninsured percentage	0.532 (−0.275 to 1.340)	—	0.418 (−0.406 to 1.242)	—
Number of providers	0.210 (−1.318 to 1.738)	—	0.458 (−1.097 to 2.012)	—
Screened	−0.002 (−0.013 to 0.009)	—	−0.003 (−0.015 to 0.008)	—
Incidence	0.101^a^ (0.006 to 0.197)	0.085^a^ (0.007 to 0.163)	—	—
Educational attainment	0.286 (−0.100 to 0.672)	0.333^b ^(0.088 to 0.578)	0.312 (−0.085 to 0.708)	0.258^a^ (0.016 to 0.499)
**AIC**	450.30	441.79	453.32	444.55
**Adjusted *R* ^2^**	0.06	0.10	0.004	0.05

Abbreviation: — , not applicable; AIC, Akaike information criterion.

a *P* = .01.

b *P* = .001.

### Standardized incidence ratio

**Figure 2:**
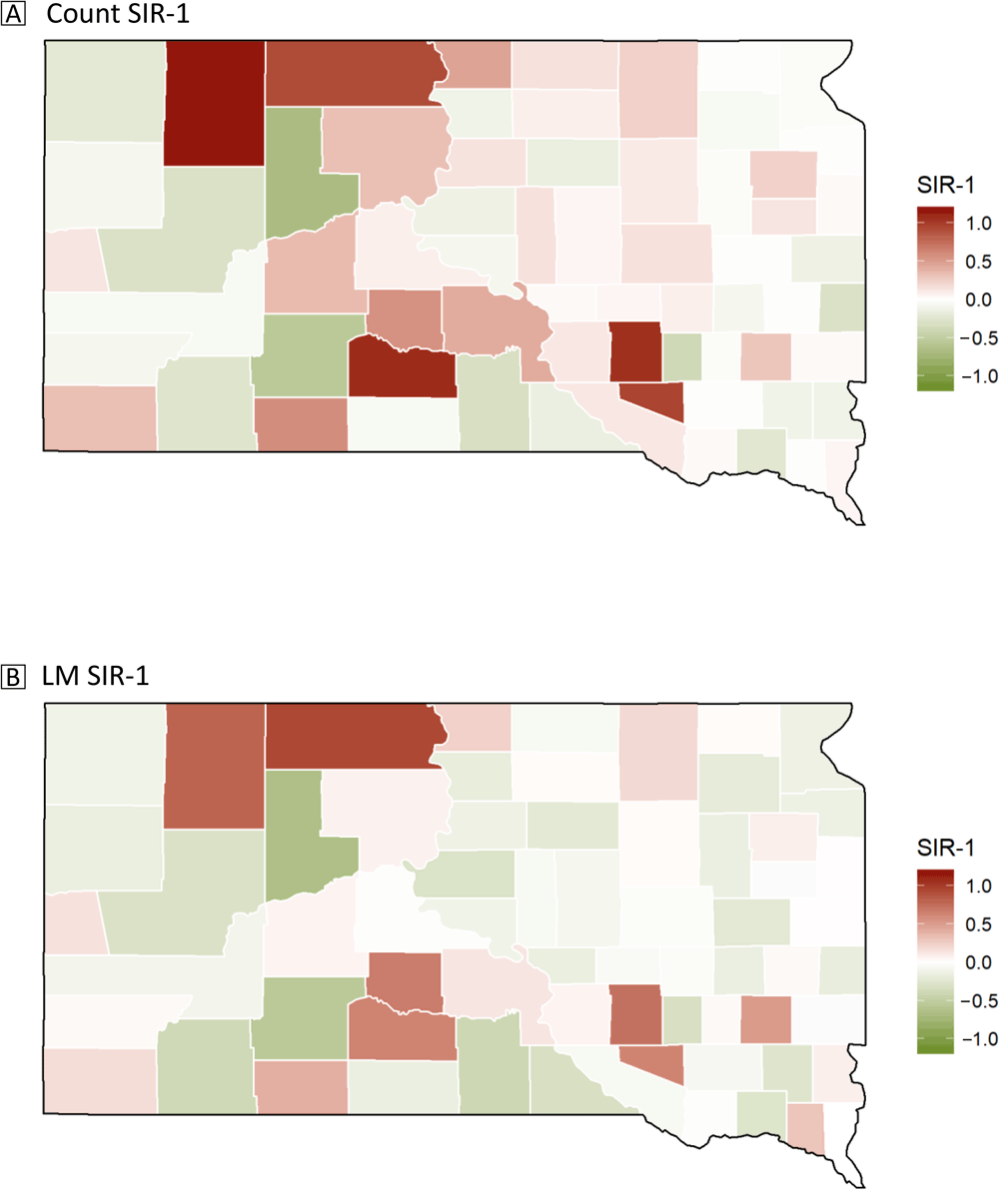
Map A shows the predicted breast cancer 2008–2017 mortality rate of South Dakota counties, accounting for age-adjustment of the data, and Map B shows the predicted breast cancer mortality rate of South Dakota counties, accounting for age-adjustment, incidence rate, and educational attainment. Abbreviation: SIR, standardized incidence ratio. Map Sources: South Dakota State Cancer Registry, South Dakota Department of Health (12), Holzhauser et al (13), and the US Census Bureau (8).

Thirty-five of the 66 counties had a *SIR_COUNT_* greater than 1, meaning that more than half of the counties had more deaths than expected (Figure 2). The 5 counties with the highest *SIR_COUNT_*, in decreasing order, were Perkins, Mellette, Aurora, Douglas, and Corson. Of those, Perkins, Mellette, and Aurora had more than twice the expected number of deaths. Ziebach, Jackson, Davison, Tripp, and Meade counties had the lowest *SIR_COUNT_*, with Ziebach and Jackson both being less than half the expected number of deaths. The highest *SIR_COUNT_* was 2.15 and the lowest was 0.31.

On the other hand, for the *SIR_LM_* most counties had fewer deaths than expected with 28 of 66 counties having a *SIR_LM_* greater than 1. The 5 counties with the highest *SIR_LM_* were similar to the *SIR_COUNT_*: Corson, Perkins, Aurora, Jones, and Mellette in decreasing order. No county had more than twice the expected number of deaths. The 5 counties with the lowest *SIR_LM_* were Ziebach, Jackson, Tripp, Oglala Lakota, and Davison. Ziebach and Jackson counties had less than half the number of expected deaths. The highest *SIR_LM_* was 1.94 and the lowest *SIR_LM_* was 0.35.

The results of the SIRs (*SIR_COUNT_* and *SIR_LM_*) are presented in Figure 2. The eastern side of the state showed similar SIR values while the western side of the state had more variation. Perkins, Mellette, Aurora, Douglas, and Corson counties had *SIR_COUNT_* values that were much higher than those of the rest of the counties. Ziebach and Jackson counties had the lowest *SIR_COUNT_* values. These counties with the highest *SIR_COUNT_* made up 5 of the 6 counties with the highest *SIR_LM_* values, with Jones County replacing Douglas County. The 2 counties with the lowest *SIR_COUNT_* values were the same 2 counties with the lowest *SIR_LM_* values.

## Discussion

Overall, we found a significant association between incidence rate and educational level with respect to breast cancer mortality rates. Breast cancer incidence was positively associated with mortality rates in South Dakota, which suggests that more breast cancer cases are associated with more breast cancer deaths. In addition, educational attainment was repeatedly identified as a significant factor for mortality. Gadeyne et al found inconclusive results in their study of breast cancer mortality and education; however, Albano et al found a significant association between educational levels and cancer in general (7,25), specifically that lower educational attainment was related to higher cancer mortality rates, reflecting the findings of this study. Race, median age, and number of women screened were not selected in the feature selection during stepwise regression in our study; similarly, race, median age, and number of women screened were not significant in our full model.

The 2020–2021 South Dakota Department of Education yearly review stated that American Indians were the largest minority group in school. However, American Indians still have a 63% completion rate for high school graduation and 59% attendance rate, compared with Whites who have a 94% completion rate for high school graduation and 94% attendance rate (26). An interesting point to consider is that South Dakota has no set standards for sex education (27). Thus, students are not taught reproductive health in general, including the importance of breast examinations, Pap smears, or prostate examinations. We advocate that set and scientifically backed health standards in high school would expose students at an early age to the risks of breast cancer and their options for screening.

The western half of South Dakota had more variability in SIR values, and the state’s demographics could be a possible explanation. The 4 counties with the highest SIRs for both count and linear model SIRs were Corson, Perkins, Aurora, and Mellette, which are either in an American Indian reservation or neighbor a county within an American Indian reservation. Research on 3 tribes in western South Dakota supported that trust is often a barrier for American Indians (as are remote location and approvals by Indian Health Service programs) (28). Research in New Mexico reported that even after in-depth implementation of screening programs that lowered the barriers of cost, availability, and access to Native American and Hispanic women, the screening rates remained low, under 40% of women annually (26). The high SIRs in or neighboring reservation counties may mean that trust is also an issue, and South Dakota has more to work on than accessibility to Native Americans.

After controlling for incidence rate and educational attainment, the *SIR_LM_* values became less variable. The SIRs’ decrease in range and mean closer to 1 indicate that the factors did affect mortality rate. This again agrees with findings from Albano et al that educational attainment affects mortality (7).

We found that some counties had a higher mortality rate than expected based on the age of the women in the county. Ziebach and Jackson counties had the highest mortality rates, and the counties with the lowest SIRs are not home to major medical centers. Haakon County is vertically between Ziebach and Jackson counties and is one of the counties that does not have a provider; however, Haakon County has a higher mortality rate than the average of counties of South Dakota and both SIRs greater than 1, which means there were more deaths than expected. The areas of the map where there are dark green counties next to dark red counties are either on an American Indian reservation or neighbor an American Indian reservation. The differences between counties do not come from any singular cause, but rather due to variations in race, poverty levels, and population size.

Our study has limitations, primarily in the absence of portions of mortality data. Because South Dakota is largely a rural state, several counties have small populations and see very few deaths from breast cancer. These numbers are then held back from the public to protect the privacy of the patients. This suppression resulted in having to impute the mortality rates of 15 counties, possibly introducing errors. The assumption of the regression model did not account for this error, which may confer bias on the results. The counties with American Indian reservations have another health system that could have resulted in the under-representation or over-representation of breast cancer deaths from those counties (29). In addition, a study found misclassification of Native Americans caused an underestimation of mortality rates as well (30). These gaps in databases and their contents highlight research challenges that rural communities will continue to face when few data are collected, populations and incidence are sparse, and data are inconsistently collected by multiple sources. Sociodemographic data are also challenging to consistently collect throughout a state. More detailed data per county would help yield accurate and unbiased results. For example, considering education, Zajacova and Lawrence argue that education is not a single-generation factor (31). Having data on the educational attainment of a patient’s parents or family, in addition to their own educational attainment, would allow us to assess the risk and see the relationship between education and breast cancer incidence or mortality. Thus, more research is needed to understand the effects education level has on financial security, stable employment, social success, and in turn, breast cancer mortality.

In conclusion, understanding the risk factors and geographic distribution of breast cancer mortality among women across the state will assist stakeholders with efforts at prevention and resource allocation guided by data.
