# Erratum, Vol. 19, October 20 Release

**DOI:** 10.5888/pcd20.220113e

**Published:** 2023-07-27

Due to author errors, the article “Spatial Analysis of Breast Cancer Mortality Rates in a Rural State” included an incorrect reporting period and inaccurate associated references. The data reporting period has been corrected from 2001–2015 to 2008–2017. The text in the article referencing the breast cancer data reporting period has been corrected as follows: “Breast cancer incidence and mortality rates from 2008 through 2017 were extracted from the South Dakota State Cancer Registry’s South Dakota Cancer County Assessment Tool (12). The tool allows access to public use cancer data. The 2008–2017 data were accessed in September 2020.”

In addition, the breast cancer data source and access date have been corrected as follows: “12. South Dakota Cancer Registry. South Dakota Cancer County Assessment Tool. Accessed September 2020. https://www.sdcancerstats.org/.” A second reference has been corrected from March 2022 to May 2021 as follows: “11. Centers for Disease Control and Prevention. National Breast and Cervical Cancer Early Detection Program screening program summaries: South Dakota. Accessed May 2021. https://www.cdc.gov/cancer/nbccedp/data/summaries/south_dakota.htm.”

The corrected article was posted on July 13, 2023, and can be found at https://www.cdc.gov/pcd/issues/2022/22_0113.htm. We regret any confusion or inconvenience these errors may have caused.

